# Optimal dietary copper requirements and relative bioavailability for weanling pigs fed either copper proteinate or tribasic copper chloride

**DOI:** 10.1186/s40104-020-00457-y

**Published:** 2020-05-22

**Authors:** Gang Lin, Yang Guo, Bing Liu, Ruiguo Wang, Xiaoou Su, Dongyou Yu, Pingli He

**Affiliations:** 1 Institute of Quality Standards and Testing Technology for Agricultural Products, Chinese Academy of Agricultural Sciences, Key Laboratory of Agrifood Safety and Quality, Ministry of Agriculture and Rural Affairs, Beijing, 10081 People’s Republic of China; 2 College of Animal Science, Zhejiang University, Key Laboratory of Animal Nutrition and Feed in East China of Ministry of Agriculture and Rural Affairs, Hangzhou Zhejiang, 310058 People’s Republic of China; 3 State Key Laboratory of Food Science and Technology, Jiangnan University, Wuxi, Jiangsu 214122 People’s Republic of China; 4 State Key Laboratory of Animal Nutrition, College of Animal Science and Technology, China Agricultural University, Beijing, 100193 People’s Republic of China

**Keywords:** Bioavailability, Copper, Copper proteinate, Excretion, Piglet, Requirement, Tribasic copper chloride

## Abstract

**Background:**

The objective of this study was to determine the effects of supplementing Cu on growth performance, Cu metabolism and Cu-related enzyme activities of weanling pigs fed diets with two different Cu sources, and to estimate optimal Cu requirements and relative bioavailability from these two sources for pigs.

**Methods:**

Weanling pigs were allocated to 14 treatments arranged factorially, including 6 added Cu levels (5, 10, 20, 40, 80, 160 mg/kg), and 2 mineral sources (tribasic Cu chloride, TBCC and copper proteinate, CuPro), as well as one negative control (0 mg/kg added Cu level) and one maximum allowed level treatment (200 mg/kg TBCC) for the entire 38-d experiment. Growth performance, mineral status and enzyme activities were measured at the end of this study.

**Results:**

Increasing levels of Cu showed linear and quadratic responses (*P <* 0.01) for final BW, ADG and FCR regardless of the sources. Supplementation with TBCC (> 80 mg/kg) and CuPro (> 20 mg/kg) significantly decreased (*P <* 0.05) diarrhea incidence of weanling pigs. There were linear and quadratic increases (*P <* 0.01) in bile, hepatic, and intestinal Cu concentrations, fecal Cu contents, and plasma enzyme activities (alkaline phosphatase, ceruloplasmin, Cu, Zn-Superoxide dismutase (Cu/Zn SOD), and glutathione peroxidase), whereas plasma malondialdehyde decreased (*P <* 0.01) linearly and quadratically as dietary Cu level increased. Similarly, pigs fed CuPro absorbed and retained more Cu and excreted less Cu than those fed TBCC when supplemented 80 mg/kg and above. Optimal dietary Cu requirements for pigs from 28 to 66 d of age estimated based on fitted broken-line models (*P <* 0.05) of bile Cu, plasma Cu/Zn SOD and growth performance were 93–140 mg/kg from TBCC, and 63–98 mg/kg from CuPro accordingly. According to slope ratios from multiple linear regression, the bioavailability value of CuPro relative to TBCC (100%) was 156–263% (*P <* 0.01).

**Conclusion:**

The findings indicated that Cu recommendation from current NRC (5–6 mg/kg) was not sufficient to meet the high requirement of weanling pigs. Cu from CuPro was significantly more bioavailable to weanling pigs than TBCC in stimulating growth and enzyme activities, decreasing diarrhea frequency and fecal Cu contents to the environment.

## Introduction

Copper (Cu) is an essential trace element for the synthesis of hemoglobin and activation of several oxidative enzymes necessary for normal metabolism and development of pigs. A minimum requirement of 5 to 6 mg/kg Cu is adequate for weanling piglets to prevent deficiency symptoms and support growth [1, 2]. Cu is provided by most feed ingredients, such as corn, wheat and soybean meal, and a basal diet without micromineral supplementation usually provides a level of Cu equal to or greater than that which is required. Whereas, in 2012 the NRC indicated that the innate minerals should be considered as “safety factors” [2], and swine industry typically feeds 10–25 mg/kg Cu for maintenance, or at pharmacological range of 100 to 250 mg/kg to provide a growth promoting effect for weanling piglets [3, 4].

Feeding pharmacological concentrations of Cu could also result in undesirable consequences in animal production. High levels of dietary Cu sulfate counteracted the utilization of other minerals, such as Zn [4], decreased apparent phosphorus retention [5] and had detrimental effects on the efficacy of phytase [6]. Of particular concern is the co-selection of Cu-tolerant and multi-antibiotics resistant in both swine [7, 8] poultry and ruminant [9], which could potentially have a negative impact on the antibiotic treatment of disease in humans and animals. Furthermore, investigators reported a 14-fold increase in fecal Cu excretion with 250 mg/kg CuSO_4_ compared with no-Cu-added basal diet [10]. The increased Cu excretion might accumulate in soil and water, resulting in reduction of crop yield [11] and potential toxicity for farm animals [12]. With respect to the potential environmental threat, different countries and regions such as China, allowed a transitional period to amend and reduce the authorized maximum Cu contents in complete feed for different target species. Therefore, it is both necessary and urgent to seek alternative Cu sources for growth stimulation which have greater bioavailability and are more environmentally friendly.

Copper may be provided either through inorganic salts, such as sulfates and oxides, or organic forms, such as chelated and Cu complexes. Cu hydroxychloride (tribasic Cu chloride, TBCC) was reported to be as effective as CuSO_4_ for growth promotion [13] but less reactive in vitamin-mineral premix due to the chloride being insoluble in water [14]. Cu proteinate (CuPro), which is the chelation of a soluble copper salt with enzymatically hydrolyzed soy protein, appeared to be more effective in enhancing the growth rate of weanling piglets, and reducing fecal Cu excretion [15] at a lower level compared with CuSO_4._ However, there is limited information regarding optimal supplementation levels of these two types of Cu for growth stimulation when fed to piglets. Furthermore, a strong correlation between growth response and Cu source bioavailability has been observed [3]. Therefore, the objective of the present study was to determine the responses of piglets to dietary Cu sources and concentrations in terms of growth performance, blood markers, bile, liver and intestinal Cu concentrations, so as to determine a series of sensitive and consistent biomarkers for the estimation of optimal dietary Cu requirement/supplementation in the forms of TBCC and CuPro after weaning (28 to 66 d of age). The relative bioavailability of organic CuPro compared with inorganic TBCC for piglets fed a practical corn-soybean meal diet was also investigated.

## Materials and methods

### Animals and housing

Crossbred weanling piglets (Duroc × Landrace × Yorkshire, *n* = 840) weaned at an average of 28 ± 3 d of age and 7.37 ± 0.49 kg body weight were randomly allotted to the treatments for the 38-day experiment based on litter and weight with sex ratio equalized within weight blocks. There were 6 replicate pens (10 pigs/pen) for each of the 14 treatments. Each identical pen (4.2 m × 3.8 m) was separated by steel with half solid concrete floors at the front and half plastic-coated expanded metal floors at the back. Pigs were always allowed ad libitum access to feed and water, via a three-hole concrete feeder and a stainless-steel water dispenser. All health pigs were fed a commercial creep feed from 14 to 28 d of age and were vaccinated with pseudorabies virus (PRV) at birth and on d 49 of age, porcine circovirus type 2 (PCV2) and *Mycoplasma hyopneumoniae* (*M*. *hyopneumoniae*) on d 14 of age, porcine reproductive and respiratory syndrome virus (PRRSV) on d 21 of age, and classic swine fever virus (CSFV) on d 28 and d 56 of age according to the manufacturer’s recommendations. Temperature was maintained and controlled by use of thermostatically controlled heaters and exhaust fans. Throughout the study, the nursery room was continuously illuminated with fluorescent lighting from 08:00 to 20:00.

### Dietary treatments

Diets were formulated to meet or exceed NRC (2012) requirements with no additional copper supplementation. The ingredients and nutrient composition of the corn-soybean-meal based diet are presented in Table 1. All diets were pelleted and contained 1,200 FTU/kg phytase (EN Bio-tech Co., Ltd., Beijing, China).

**Table 1:** Ingredient and calculated nutrient composition of basal diet, as-fed basis^a^

Ingredient	Content, %	Calculated nutrient value	
Ground corn	38	DE, Mcal/kg	3.42
Expanded corn	20	CP, %	18.70
Soybean meal	8	EE, %	3.75
Fermented soybean meal	10	Ca, %	0.91
Extruded soybean	7	P, %	0.68
Fishmeal	3	Av. P, %	0.50
Whey powder	6	Lys, %	1.29
Glucose	2	Thr, %	0.82
Soybean oil	2	Met, %	0.38
Calcium citrate	1		
Dicalcium phosphate	1		
Premix^1^	2		
Total	100		

^a^ Provided per kilogram of diet: vitamin A, 14,000 IU; vitamin D 2,800 IU; vitamin E, 100 IU; vitamin K, 4 mg; vitamin B_1_, 4 mg; vitamin B_2_, 10 mg, vitamin B_6_, 6 mg, vitamin B_12_, 60 μg, biotin, 300 μg, folic acid, 15 mg, pantothenic acid, 40 mg; 0.6 mg I as Ca (IO_3_)_2_; 150 mg Fe as FeSO_4_; 40 mg Mn as MnSO_4_; 800 mg Zn as ZnO; 0.4 mg Se as Na_2_SeO_3_; and 1,200 FTU/kg phytase. Different sources of copper replaced the cornstarch in premix to create different dietary treatments containing 0, 5, 10, 20, 40, 80, 160 and 200 mg/kg Cu

A completely randomized design involving a 2 × 6 factorials arrangement of treatments plus one negative control and one maximum level treatment were used in this experiment. Two supplementation copper sources: inorganic; copper hydroxychloride (Tribasic Cu Chloride, TBCC, 58% Cu, Shenzhen Luhuan Chemical Co., Ltd., China), and organic; copper proteinate (Cu-Pro, 10% Cu, Bioplex® Cu, Alltech Inc., Nicholasville, KY, USA) were used. NRC recommended 5–6 mg/kg Cu for weanling pigs. Therefore, the six supplemental copper levels were 5 10, 20, 40, 80 and 160 mg of Cu/kg of diet. The negative control (NC) had no Cu added to the basal diet. The last treatment was basal diet plus 200 mg/kg (maximum level of copper allowance in China, MARA regulation No. 1224) Cu from TBCC. Feed samples were obtained for determination of copper concentrations.

### Performance measurements and sample collection

Piglet weights and feed consumption were measured by pens at the beginning and at end of the experiment to determine ADG (average daily gain), ADFI (average daily feed intake) and FCR (feed conversion ratio). Diarrhea scores were assessed visually every 4 d until 36 d of the experiment by 2 independent observers via a score from 1 to 5 (5 = watery diarrhea, 4 = severe diarrhea, 3 = mild diarrhea, 2 = moist feces, and 1 = normal feces). Frequency of diarrhea was calculated by adding all days for a pig with a score of 3 or greater.

Frequency = numbers of pigs with diarrhea / (numbers of pigs × the number of days assessing diarrhea scores) × 100%.

During the last 3 d of experiment, fecal grab samples were collected from three randomly selected pigs per pen by entering the pen and waiting until pigs voluntarily passed. Fecal samples were stored in plastic freezer bags, and immediately frozen at − 20 °C. The 3-day fecal collections from individual pens were thawed, pooled, and then dried in an oven at 65 °C for 48 h.

All pigs were weighed on d 38 of the trial. After weighing, three random medium-size pigs per treatment (NC, 20, 40, 80 and 160 mg/kg of TBCC and CuPro, and 200 mg/kg TBCC) were placed in a separate room and harvested. The remaining pigs were then fed with the same experimental diets for an extra day. Another three pigs per treatment were harvested on d 39. Therefore, one pig per pen was slaughtered at the end. After fasting for 12 h, a 10 mL blood sample was taken via anterior cava vena from selected pig per pen into a heparinized Vacutainer tube (Kangjie Medical Devices Co., Ltd., Jiangsu, China), containing 100 USP units of Na heparin. The blood was then centrifuged at 2,500×*g* for 20 min at 4 °C and the plasma samples were collected and stored in labelled microfuge tubes at − 20 °C until analysis. Afterward, these selected pigs were anesthetized by electric shock and then humanely euthanized via exsanguination. Subsequently, the abdominal cavity was lacerated vertically to collect bile, liver, and intestinal samples from the duodenum and proximal jejunum. Each section of the intestine was then opened and rinsed with 1× PBS to clean the intestinal epithelium of digesta and other debris. Subsequently, mucosal scrapings were collected from each section using the method described by Hansen et al. [16]. All tissue samples were immediately stored in liquid nitrogen and then stored at − 80 °C before analysis.

### Sample mineral analysis

Concentrations of Cu in feed (on an as-fed basis) were determined from triplicate samples of each dietary treatment. Pooled tap water was also tested for water Cu supply. Feed and fecal samples were finely ground to pass through a 1-mm screen; liver and intestinal samples were rinsed with double-distilled water, freeze-dried, finely ground via a 1-mm screen to produce homogenates, and subsequently extracted fat with diethyl ether (method 963.15; AOAC, 2000) [17]. Plasma was deproteinated by 20% (w/v) trichloroacetic acid and the supernatant fraction obtained. Bile samples were treated for subsequent analysis as described by Armstrong et al. [18]. The levels of copper, zinc and iron in diets, plasma, feces, liver, duodenal, and jejunal mucosa were measured with a flame atomic absorption spectrophotometer (model Thermo Scientific S Series, Thermo Fisher Scientific Inc., USA), after wet-washing preparation with nitric acid and hydrogen peroxide (method 999.10; AOAC, 2000) [17]. Validation of the mineral analysis was conducted concurrently using bovine liver powder [GBW (E) 080193; National Institute of Standards and Technology, Beijing, China] as a standard reference material (SRM). Dietary Cu concentrations were double-verified by an inductively coupled plasma optical emission spectrometer (ICP-OES, OPTIMATM 2100 DV, Perkin Elmer Inc., Shelton, CT, USA). The dietary, fecal and tissue concentrations of minerals are expressed as mg/kg, and water, plasma and bile concentrations are expressed as μg/mL.

### Plasma enzyme activity measurement

The procedures outlined in a previous study [19] were followed to extract and test the activity of protein and enzymes. Exactly 0.3 g of liver samples we weighed and homogenized in cold 0.1 mol/L Tris-HCl buffer at pH 7.4 to produce a 10% (w/v) homogenate. After centrifugation for 10 min at 10,000×*g* at 4 °C, the supernatant was collected for measurement of protein and specific enzyme activities. Plasma parameters were measured using commercial assay kits according to the manufacturer’s instructions (Nanjing Jiancheng Bioengineers Institute, China). Alkaline phosphatase (ALP), ceruloplasmin (CER), glutathione peroxidase (GSH-Px), and total antioxidant capability (T-AOC) were determined by the colorimetric method, while Cu, Zn-superoxide dismutase (Cu/Zn SOD), and malondialdehyde (MDA) were subjected to the xanthine oxidase method and thiobarbituric acid reactive substances (TBARS) assay, respectively. The intra and inter CVs were 3.5% and 4.1% for ALP; 2.7% and 5.3% for CER; 3.6% and 6.8% for GSH-Px; 3.6% and 6.4% for T-AOC; 3.3% and 5.5% for Cu/Zn SOD; and 2.3% and 5.3% for MDA, respectively. All plasma enzyme activities are expressed as units per milliliter.

### Statistical analysis

Data were analyzed as a randomized complete block design using the MIXED model procedure of SAS version 9.2. The pen averages served as the experimental unit for growth performance and fecal mineral concentrations, while the individual pig (one pig per pen) served as the experimental unit for tissue mineral contents and enzyme activities. Sampling day was also considered as a block when analyzed tissue and plasma parameters. There was no significant difference in these data between two sampling days. Data excluding the negative control and 200 mg/kg TBCC group were analyzed as a factorial (source × level) arrangements of treatments by two-way ANOVA using the GLM procedure of SAS. The model included the effects of Cu source, added Cu level and their interaction. To evaluate the effect of two sources of copper supplementation, data from either TBCC or CuPro group were subjected to one-way ANOVA separately using the GLM procedure from SAS version 9.2. Another ANOVA test was introduced to compared differences among all treatments. Differences in means among treatments were tested using the Tukey HSD method. Orthogonal comparisons were applied for linear and quadratic responses of dependent variables to added Cu levels. Furthermore, a paired t-test was employed to compare all values between TBCC and CuPro groups on each inclusion level. Regression analyses of broken-line (2 straight-line, one-breakpoint) model were performed to estimate Cu optimization (the break point from a broken-line model) using the best fit between responsive criteria and dietary Cu levels. Relative bioavailability values of Cu as CuPro were estimated by slope ratio comparison based on multiple linear regressions using TBCC as the standard source [20, 21]. The regressions were calculated using added Cu daily intake (adjusted by feed intake) as the independent variable rather than added Cu level [22]. Slope ratio and standard error (SE) were estimated using the method of error propagation as described by Littell et al. [23]. The difference between the two Cu sources was determined according to the difference respective regression coefficients. The level of statistical significance was set at *P <* 0.05, while 0.05 < *P* < 0.10 was considered as a positive trend for significance.

## Results

The analyzed values for Cu agreed with the calculated ones. Copper concentrations in all diets are presented in Table 2. The basal diet contained 9.4 mg/kg of Cu, and tap water Cu concentration was 0.3 mg/L.

**Table 2:** Analyzed copper contents in the experimental diets (mg/kg, as-fed basis)

Copper source^1^	Copper level, mg/kg							
	0	5	10	20	40	80	160	200
TBCC	9.4 ± 2.1	14.9 ± 3.0	21.9 ± 3.3	28.1 ± 3.2	50.4 ± 4.7	90.6 ± 5.2	174.2 ± 10.4	213.4 ± 14.6
CuPro	9.4 ± 2.1	15.4 ± 2.2	22.3 ± 3.8	30.8 ± 3.5	51.3 ± 5.2	88.7 ± 9.8	170.8 ± 13.4	–

Value expressed as mean ± standard deviation (SD) based on triplicate determinations

^1^*TBCC* tribasic copper chloride, *CuPro* copper proteinate

### Growth performance

Copper Supplementation did not affect (*P >* 0.05) ADFI but did affect ADG (*P <* 0.05) and FCR (*P <* 0.001) for both Cu sources (Table 3 and S1). Weight gain increased linearly (*P <* 0.01) and quadratically (*P <* 0.001) as dietary TBCC and CuPro concentration increased. There was no further increase in ADG when dietary Cu supplementation was more than 160 mg/kg Cu as TBCC, or 80 mg/kg Cu as CuPro. The FCR decreased linearly (*P =* 0.001) for TBCC groups and decreased (*P <* 0.001) both linearly and quadratically for CuPro groups as Cu levels increased in the diets. Greater (*P <* 0.05) ADG was observed for pigs fed diets with CuPro compared with pigs fed with TBCC at the level of 80 mg/kg only, while improved FCR (*P <* 0.05) was observed for pigs fed CuPro compared with the TBCC at the levels from 10 to 80 mg/kg, resulting in higher (*P <* 0.05) final body weight in pigs receiving CuPro than those receiving TBCC as a copper source at the levels of 40 and 80 mg/kg diet.

**Table 3:** Effect of amount and source of dietary copper on growth performance of weanling pigs

	Copper source^1^	Copper level, mg/kg								Pooled SEM	*P*-value		
		0	5	10	20	40	80	160	200		Treatment	Linear	Quadratic
BW d 0, kg	TBCC	7.30	7.43	7.40	7.43	7.43	7.23	7.27	7.33	0.30	0.999	0.678	0.892
	CuPro	7.30	7.41	7.44	7.37	7.47	7.33	7.45	–	0.37	0.999	0.865	0.984
BW d 38, kg	TBCC	18.66	18.83	18.91	18.95	19.48	19.82	20.88	20.87	0.83	0.362	0.003	0.013
	CuPro	18.66	18.87	19.01	19.24	19.97^*^	21.18^*^	21.13	–	0.79	0.166	0.004	0.006
ADG, g	TBCC	298.9^b^	300.1^b^	302.8^b^	303.2^b^	317.2^b^	331.2^ab^	358.1^a^	356.2^a^	7.78	0.032	0.003	< 0.001
	CuPro	298.9^b^	301.5^b^	304.4^b^	312.4^b^	328.9^b^	364.6^a, *^	359.9^a^	–	6.80	0.002	< 0.001	< 0.001
ADFI, g	TBCC	492.6	497.4	501.5	494.8	500.0	504.5	513.0	519.9	21.68	0.985	0.218	0.475
	CuPro	492.6	494.4	492.9	497.6	502.3	518.1	513.1	–	10.65	0.491	0.144	0.069
FCR	TBCC	1.65^ab^	1.66^a^	1.66^a^	1.63^ab^	1.58^bc^	1.52^cd^	1.43^e^	1.46^de^	0.02	<0.001	0.001	0.111
	CuPro	1.65^a^	1.64^a^	1.62^ab, *^	1.59^b, *^	1.53^c, *^	1.42^d, *^	1.43^d^	–	0.01	<0.001	< 0.001	< 0.001

^a-d^ Means within a row without a common superscript letter are different (*P <* 0.05)

* different from the corresponding TBCC group at the same supplemented copper level, *P <* 0.05

^1^*TBCC* tribasic copper chloride, *CuPro* copper proteinate

A reduction (*P <* 0.05) in diarrhea frequency was observed when pig diets were supplemented with more than 80 mg/kg of TBCC or 40 mg/kg of CuPro (Table 4). Quadratic effects (*P <* 0.05) and linear effects (*P <* 0.05) on diarrhea incidence reduction are exhibited for TBCC and CuPro supplementation, respectively.

**Table 4:** Frequency of diarrhea^1^ (%) of weanling pigs fed diets with different amount and source of copper

Copper source^2^	Copper level, mg/kg								Pooled SEM	*P*-value		
	0	5	10	20	40	80	160	200		Treatment	Linear	Quadratic
TBCC	43.6^a^	44.0^a^	43.4^a^	41.6^a^	38.4^ab^	35.8^b^	36.0^b^	35.2^b^	2.02	0.035	0.153	0.021
CuPro	43.6^a^	43.0^a^	44.0^a^	40.0^b^	39.8^b^	32.8^c,*^	35.2^c^	–	2.24	0.015	0.037	0.168

^a-c^ Means within a row without a common superscript letter are different (*P <* 0.05)

^*^ different from the corresponding TBCC group at the same supplemented copper level, *P <* 0.05

^1^ Diarrhea score = 5, watery diarrhea, 4, severe diarrhea, 3, mild diarrhea, 2, moist feces, and 1, normal feces. Frequency of diarrhea was calculated by adding all days for a pig with a score of 3 or greater. Frequency = numbers of pigs with diarrhea / (numbers of pigs × the number of days assessing diarrhea scores) × 100%, the number of days assessing diarrhea scores = 9

^2^*TBCC*  tribasic copper chloride, *CuPro*  copper proteinate

### Cu concentrations in biofluid and tissues

Plasma Cu levels were not affected (*P >* 0.05) by either dietary Cu level or source (Tables 5 and S2). However, bile Cu concentrations, hepatic and intestinal mucosal Cu retention were affected (*P <* 0.001) by levels of dietary Cu which increased linearly (*P <* 0.001) and quadratically (*P <* 0.001) as dietary Cu concentration increased, and differed between both Cu sources (*P <* 0.05). Bile Cu increased (*P <* 0.001) dramatically when pigs received more than 40 mg/kg of Cu as TBCC, while a significant response (*P <* 0.001) was observed for pigs receiving more than 20 mg/kg of Cu as CuPro. Cu concentration in liver, duodenal and jejunal mucosa differed (*P <* 0.001) markedly, when dietary Cu supplementation was more than 160 mg/kg as TBCC or 80 mg/kg as CuPro.

**Table 5:** Effect of amount and source of dietary copper on plasma, bile, intestinal, and liver copper concentrations of weanling pigs

	Copper source^1^	Copper level, mg/kg						Pooled SEM	*P*-value		
		0	20	40	80	160	200		Treatment	Linear	Quadratic
Plasma, μg/mL	TBCC	1.40	1.41	1.41	1.39	1.38	1.36	0.02	0.320	0.029	0.074
	CuPro	1.40	1.42	1.40	1.39	1.38	–	0.02	0.390	0.144	0.350
Bile, μg/mL	TBCC	0.92^d^	0.89^d^	1.95^c^	2.87^b^	3.23^a^	3.20^a^	0.03	< 0.001	< 0.001	< 0.001
	CuPro	0.92^e^	1.10^d, *^	2.36^c, *^	3.21^b, #^	3.44^a, *^	–	0.04	< 0.001	< 0.001	< 0.001
Liver, mg/kg	TBCC	23.43^b^	23.58^b^	24.12^b^	24.00^b^	32.96^a^	33.92^a^	0.61	< 0.001	< 0.001	< 0.001
	CuPro	23.43^c^	23.58^c^	23.77^c^	31.91^b, *^	37.01^a, *^	–	0.41	< 0.001	< 0.001	< 0.001
Duodenum, mg/kg	TBCC	2.80^bc^	2.77^c^	2.80^bc^	2.89^b^	3.41^a^	3.38^a^	0.02	< 0.001	< 0.001	< 0.001
	CuPro	2.80^c^	2.80^c^	2.83^c^	3.06^b, *^	3.58^a, *^	–	0.02	< 0.001	< 0.001	< 0.001
Jejunum, mg/kg	TBCC	5.01^c^	4.97^c^	5.05^bc^	5.07^bc^	5.15^b^	5.28^a^	0.03	< 0.001	< 0.001	< 0.001
	CuPro	5.01^c^	5.09^bc, *^	5.10^bc^	5.12^b, *^	5.30^a, *^	–	0.02	< 0.001	< 0.001	< 0.001

^a-d^ Means within a row without a common superscript letter are different (*P <* 0.05)

* different from the corresponding TBCC group at the same supplemented copper level, *P <* 0.05

^#^ tend to be different from the corresponding TBCC group at the same supplemented copper level, 0.05 < *P <* 0.10

^1^*TBCC* tribasic copper chloride, *CuPro* copper proteinate

Compared with pigs fed with TBCC, those fed CuPro had higher (*P <* 0.05) bile Cu contents from 20 mg/kg of Cu, and had greater (*P <* 0.05) Cu concentration in liver, duodenum and jejunum from 80 mg/kg of Cu. There was also a significant difference (*P <* 0.05) in jejunal Cu concentration at the level of 20 mg/kg in pigs fed with CuPro compared with those fed with TBCC.

### Zn and Fe concentrations in biofluid and tissues

Zn concentrations in plasma were not affected by level or source of dietary Cu (Tables 6 and S3). There were both linearly (*P <* 0.001) and quadratically (*P =* 0.0023) reduction on liver Zn contents in CuPro group, however, pigs fed TBCC at levels of 160 and 200 mg/kg had lower (*P <* 0.001) hepatic Zn compared with those fed lower levels, which decreased both linearly (*P =* 0.009) and quadratically (*P =* 0.037) as dietary TBCC concentration increased. Duodenal Zn concentration decreased both linearly (*P <* 0.001) and quadratically (*P <* 0.001) when pigs fed increasing levels of TBCC, but did not differ (*P* > 0.05) in pigs fed with different levels of CuPro. No significant differences (*P* > 0.05) were observed in jejunal Zn concentration in pigs fed different levels of either TBCC or CuPro.

**Table 6:** Effect of amount and source of dietary copper on plasma, intestinal, and liver Zn and Fe concentrations of weanling pigs

	Copper source^1^	Copper level, mg/kg						Pooled SEM	*P*-value		
		0	20	40	80	160	200		Treatment	Linear	Quadratic
Plasma Zn, μg/mL	TBCC	1.63	1.63	1.65	1.64	1.67	1.66	0.02	0.509	0.066	0.170
	CuPro	1.63	1.66	1.67	1.70	1.67	–	0.03	0.482	0.265	0.165
Liver Zn, mg/kg	TBCC	315.96^a^	318.83^a^	310.89^a^	309.09^ab^	287.04^b^	288.11^b^	4.96	< 0.001	< 0.001	< 0.001
	CuPro	315.96	317.92	316.48	313.83	305.50^*^	–	4.16	0.379	< 0.001	0.023
Duodenum Zn, mg/kg	TBCC	109.51	108.66	110.67	110.24	110.56	111.71	0.87	0.068	0.009	0.037
	CuPro	109.51	111.09	108.41	110.08	111.12	–	1.05	0.362	0.345	0.529
Jejunum Zn, mg/kg	TBCC	113.34	114.16	114.79	114.25	114.73	115.73	0.94	0.626	0.106	0.279
	CuPro	113.34	116.69^*^	115.64^*^	115.98^*^	116.22^#^	–	1.05	0.241	0.262	0.303
Plasma Fe, μg/mL	TBCC	4.16	4.12	4.17	4.27	4.25	4.34	0.17	0.945	0.314	0.606
	CuPro	4.16	4.24	4.21	4.27	4.23	–	0.13	0.988	0.770	0.876
Liver Fe, mg/kg	TBCC	368.19	365.71	362.06	361.25	375.93	374.49	7.80	0.682	0.194	0.299
	CuPro	368.19	360.99	366.26	372.85	372.71	–	12.25	0.954	0.550	0.838
Duodenum Fe, mg/kg	TBCC	249.15	248.53	248.02	246.48	245.72	246.92	1.13	0.291	0.040	0.042
	CuPro	249.15	245.83^*^	246.59	245.59	246.44	–	1.26	0.323	0.348	0.206
Jejunum Fe, mg/kg	TBCC	140.26	140.53	139.83	140.47	140.65	140.18	0.27	0.368	0.683	0.830
	CuPro	140.26	139.29	140.65^*^	140.37	140.48	–	0.60	0.548	0.475	0.758

^a-b^ Means within a row without a common superscript letter are different (*P <* 0.05)

* different from the corresponding TBCC group at the same supplemented copper level, *P <* 0.05

^#^ tend to be different from the corresponding TBCC group at the same supplemented copper level, 0.05 < *P <* 0.10

^1^*TBCC* tribasic copper chloride, *CuPro* copper proteinate

The liver Zn concentration of pigs fed with 160 mg/kg of CuPro was greater (*P <* 0.05) than pigs fed 160 mg/kg of TBCC. Proximal jejunal Zn content was higher in CuPro pigs compared with TBCC pigs at the supplementation levels of 20, 40 and 80 mg/kg, respectively; and tended to be greater (0.05 < *P* < 0.1) at the level of 160 mg/kg.

Fe concentrations in plasma, liver, duodenal and jejunal mucosa did not differ (*P >* 0.05) among different dietary Cu levels in either Cu sources. Plasma and hepatic Fe concentrations were not affected (*P >* 0.05) by dietary Cu sources. Mucosal Fe concentration was higher (*P <* 0.05) in duodenum of TBCC pigs when supplemented with 20 mg/kg Cu, whereas concentration was lower (*P <* 0.05) in jejunum with supplementation of 40 mg/kg Cu, compared with those of CuPro pigs.

### Fecal Cu, Zn and Fe concentrations

Regardless of the sources, fecal Cu concentrations increased both linearly (*P <* 0.001) and quadratically (*P <* 0.001) with increasing dietary Cu levels (Table 7 and S4). Differences (*P <* 0.05) in fecal Cu concentrations were observed between the two Cu sources from 20 to 160 mg/kg Cu, respectively. Dietary Cu levels and sources did not affect (*P >* 0.05) Zn and Fe concentrations in the feces.

**Table 7:** Effect of amount and source of dietary copper on fecal micromineral concentrations of weanling pigs (mg/kg, as air-dry basis)

	Copper source^1^	Copper level, mg/kg						Pooled SEM	*P*-value		
		0	20	40	80	160	200		Treatment	Linear	Quadratic
Fecal Cu	TBCC	121.30^f^	280.10^e^	415.37^d^	656.00^c^	917.14^b^	1353.44^a^	8.49	< 0.001	< 0.001	< 0.001
	CuPro	121.30^e^	255.82^d, *^	362.15^c, *^	600.67^b, *^	873.10^a, *^	–	7.22	< 0.001	< 0.001	< 0.001
Fecal Zn	TBCC	5720.19	5865.70	5729.98	5885.98	5786.74	5899.92	62.16	0.198	0.190	0.414
	CuPro	5720.19	5928.12	5760.37	5883.88	5793.44	–	118.13	0.711	0.920	0.776
Fecal Fe	TBCC	3058.96	3051.32	2998.86	2989.07	2953.81	3042.00	98.57	0.963	0.685	0.668
	CuPro	3058.96	2963.80	2964.34	3000.19	2976.71	–	57.77	0.754	0.605	0.740

^a-f^ Means within a row without a common superscript letter are different (*P <* 0.05)

* different from the corresponding TBCC group at the same supplemented copper level, *P <* 0.05

^1^*TBCC* tribasic copper chloride, *CuPro* copper proteinate

### Plasma enzyme activity

As shown in Tables 8 and S5, plasma T-AOC levels were not affected by level or source of dietary Cu (*P >* 0.05). The activities of ALP, Ceruloplasmin, Cu/Zn SOD, GSH-Px in plasma increased linearly (*P <* 0.01) and quadratically (*P <* 0.01) as dietary Cu concentration increased regardless of the sources. However, MDA level decreased linearly (*P <* 0.001) and quadratically (*P <* 0.001) with increasing dietary Cu level in both Cu sources.

**Table 8:** Effect of amount and source of dietary copper on plasma enzyme activities and malondialdehyde (MDA) concentrations of weanling pigs

	Copper source^1^	Copper level, mg/kg						Pooled SEM	*P*-value		
		0	20	40	80	160	200		Treatment	Linear	Quadratic
ALP, U/100 mL	TBCC	1.68^d^	1.78^d^	2.05^c^	2.36^b^	2.94^a^	3.00^a^	0.06	< 0.001	< 0.001	< 0.001
	CuPro	1.68^e^	1.91^d^	2.25^c, *^	2.80^b, *^	3.05^a^	–	0.05	< 0.001	< 0.001	< 0.001
CER, U/mL	TBCC	57.34^e^	73.12^d^	76.26^cd^	80.12^bc^	83.27^ab^	85.90^a^	1.05	< 0.001	< 0.001	< 0.001
	CuPro	57.34^c^	78.91^b, *^	83.67^ab, #^	85.35^ab, *^	88.74^a, *^	–	2.13	< 0.001	< 0.001	< 0.001
Cu/Zn SOD, U/mL	TBCC	121.76^b^	135.09^ab^	134.96^ab^	139.82^ab^	146.71^a^	145.34^a^	5.23	0.026	0.002	0.003
	CuPro	121.76^c^	133.97^bc^	140.68^abc^	149.85^ab, *^	154.82^a^	–	4.99	< 0.001	< 0.001	< 0.001
GSH-Px, U/mL	TBCC	730.31^c^	729.40^c^	741.17^b^	750.61^a^	748.72^a^	746.85^a^	1.18	< 0.001	< 0.001	< 0.001
	CuPro	730.31^c^	732.63^bc, #^	738.90^b^	756.35^a^	757.54^a, *^	–	1.87	< 0.001	< 0.001	< 0.001
MDA, nmol/mL	TBCC	2.36^a^	2.31^ab^	2.17^abc^	1.81^bc^	1.78^c^	1.84^bc^	0.12	0.002	< 0.001	< 0.001
	CuPro	2.36^a^	2.11^ab^	1.89^bc, *^	1.79^bc^	1.75^c^	–	0.08	< 0.001	< 0.001	< 0.001
T-AOC, U/mL	TBCC	0.61	0.62	0.63	0.69	0.71	0.71	0.06	0.706	0.100	0.233
	CuPro	0.61	0.65	0.66	0.71	0.72	–	0.04	0.442	0.072	0.148

^a-c^ Means within a row without a common superscript letter are different (*P <* 0.05)

* different from the corresponding TBCC group at the same supplemented copper level, *P <* 0.05

^#^ tend to be different from the corresponding TBCC group at the same supplemented copper level, 0.05 < *P <* 0.10

^1^*TBCC* tribasic copper chloride, *CuPro* copper proteinate

*ALP* alkaline phosphatase, *CER* ceruloplasmin, *Cu/Zn SOD* Cu, Zn-superoxide dismutase, *GSH-Px* glutathione peroxidase, *MDA* malondialdehyde, *T-AOC* total antioxidant capability

Significant Cu source effects (*P <* 0.05) were observed at different dietary Cu levels depending on the parameters tested. ALP levels were higher (*P <* 0.05) for pigs fed 40 or 80 mg/kg Cu in the form of CuPro; CER concentrations were higher (*P <* 0.05) for pigs fed 20, 80, or 160 mg/kg Cu in the form of CuPro, compared with corresponding levels of TBCC. Compared with CuPro pigs, TBCC pigs had lower (*P <* 0.05) Cu/Zn SOD activity when supplemented with 80 mg/kg Cu, and lower (*P <* 0.05) GSH-Px activity when fed 160 mg/kg Cu, but had higher (*P <* 0.05) MDA concentration with 40 mg/kg Cu. In addition, a reduction trend was observed in TBCC pigs for CER (*P =* 0.064) when fed with 40 mg/kg Cu or GSH-Px (*P =* 0.083) with 20 mg/kg Cu.

### Optimal supplementation cu requirement

Results of optimal supplementation dietary Cu levels either in the form of TBCC or CuPro as estimated by the broken-line model are presented in Table 9. Based on the fitted models of ADG and FCR, activities in plasma Cu/Zn SOD, CER, GSH-Px, ALP, MDA, and bile Cu concentration, optimal supplementation dietary Cu levels were 50–148 mg/kg as TBCC and 27–98 mg/kg as CuPro for piglets fed a corn and soybean-meal diet after weaning from 28 to 66 d of age (BW 7–20 kg). Concentrations of hepatic and duodenal Cu were fitted with logistic regression and were not suitable for broken line analysis. Jejunal Cu concentration increased linearly as dietary Cu increased and was not suitable for estimating the Cu requirement of pigs, but could be used to study the bioavailability of different Cu sources for pigs.

**Table 9:** Estimation of dietary Cu requirements for weanling pigs fed diets with TBCC or CuPro based on broken-line regression analysis

Dependent variables	Copper source^1^	Regression equation^2^	RMSE^3^	Dietary Cu requirement^4^, mg/kg
ADG	TBCC	*Y* = 357.15–0.420 × (*X* < 140.65) × (140.65 − *X*)	18.03	140
	CuPro	*Y* = 361.37–0.800 × (*X* < 80.00) × (80.00 − *X*)	30.20	80
FCR	TBCC	*Y* = 1.445 + 0.0018 × (*X* < 119.93) × (119.93 − *X*)	0.023	120
	CuPro	*Y* = 1.423 + 0.0030 × (*X* < 73.81) × (73.81 − *X*)	0.022	74
ALP	TBCC	*Y* = 2.973–0.009 × (*X* < 148.33) × (148.33 −*X*)	0.144	148
	CuPro	*Y* = 3.048–0.014 × (*X* < 97.55) × (97.55 − *X*)	0.122	98
Bile Cu	TBCC	*Y* = 3.216–0.0267 × (*X* < 93.38) × (93.38 − *X*)	0.207	93
	CuPro	*Y* = 3.228–0.0360 ×(*X* < 69.10) × (69.10 − *X*)	0.269	69
CER	TBCC	*Y* = 84.58–0.252 × (*X* < 50.00) × (50.00 − *X*)	3.73	50
	CuPro	*Y* = 85.92–1.079 × (*X* < 26.50) × (26.50 − *X*)	5.32	27
Cu/Zn SOD	TBCC	*Y* = 146.03–0.194 × (*X* < 102.67) × (102.67 − *X*)	12.58	103
	CuPro	*Y* = 152.33–0.473 × (*X* < 62.72) × (62.72 − *X*)	11.94	63
GSH-Px	TBCC	*Y* = 748.73–0.272 × (*X* < 75.59) × (75.59 − *X*)	3.71	76
	CuPro	*Y* = 757.54–0.337 × (*X* < 88.37) × (88.37 − *X*)	4.84	88
MDA	TBCC	*Y* = 1.81 + 0.0070 × (*X* < 84.87) × (84.87 − *X*)	0.279	85
	CuPro	*Y* = 1.77 + 0.0116 × (*X* < 49.80) × (49.80 − *X*)	0.20	50
T-AOC	TBCC	*Y* = 0.709–0.0010 × (*X* < 105.12) × (105.12 − *X*)	0.141	105
	CuPro	*Y* = 0.711–0.0012 × (*X* < 77.67) × (77.67 – *X*)	0.101	78

^1^*TBCC*  tribasic copper chloride, *CuPro* copper proteinate

^2^ Y is the dependent variable and X is the supplementation Cu concentration in the corresponding diet (mg/kg)

^3^ RMSE: root mean squared error

^4^ Dietary Cu requirement = Supplemental Cu level

*ALP* alkaline phosphatase, *CER* ceruloplasmin, *Cu/Zn SOD* Cu, Zn-superoxide dismutase, *GSH-Px* glutathione peroxidase, *MDA* malondialdehyde, *T-AOC* total antioxidant capability

### Relative bioavailability estimates of CuPro

Due to a lack of data on natural forms of Cu, regressions were calculated based on daily dietary added Cu intake during the experimental period, rather than added Cu level or analyzed total Cu intake (Table 10) within the linear range as indicated by broken-line analysis. Significant (*P* < 0.01) multiple linear regression relationships were observed in the ADG and FCR activities of plasma Cu/Zn SOD, CER, GSH-Px, MDA and ALP, as well as jejunal Cu concentrations. Therefore, the relative bioavailability values were estimated based on these parameters of daily dietary added Cu intake (Table 11). Significant (*P* < 0.01) differences in slopes between TBCC and CuPro were found in ADG, FCR, plasma CER and ALP activities, and jejunal Cu concentration. When the response to TBCC was set to 100%, the estimated relative bioavailability of CuPro was 183%, 172%, 263%, 156% and 208% based on the indices mentioned above, respectively, indicating greater availability for enhancing or improving these corresponding parameters when compared with TBCC.

**Table 10:** Multiple linear regressions of ADG, FCR, plasma enzyme activities, and jejunal Cu concentrations on daily analyzed dietary Cu intake^1^

Dependent variables	Regression equation^2^	R^2^	*P*-value
ADG	*Y* = 296.113 + 0.8916*X*_*1*_ + 1.63437*X*_*2*_	0.61	< 0.0001
FCR	*Y* = 1.662–0.003535*X*_*1*_ − 0.006068*X*_*2*_	0.92	< 0.0001
ALP	*Y* = 1.629 + 0.018343*X*_*1*_ + 0.028563*X*_*2*_	0.88	< 0.0001
CER	*Y* = 74.487 + 0.11645*X*_*1*_ + 0.306*X*_*2*_	0.79	< 0.0001
Cu/Zn SOD	*Y* = 131.045 + 0.2185*X*_*1*_ + 0.4489*X*_*2*_	0.32	0.0019
GSH-Px	*Y* = 724.255 + 0.6748*X*_*1*_ + 0.7661*X*_*2*_	0.86	< 0.0001
MDA	*Y* = 2.326–0.011156*X*_*1*_ − 0.014865*X*_*2*_	0.37	0.0005
Jejunal Cu	*Y* = 5.004 + 0.00168*X*_*1*_ + 0.00349*X*_*2*_	0.77	< 0.0001

^1^ Daily dietary analyzed Cu intake = ADFI times analyzed supplemental dietary Cu content (analyzed total Cu content – background Cu content) for each respective Cu source

^2^ X_1_ is the analyzed daily added Cu intake (mg) from tribasic copper chloride, and X_2_ is the analyzed daily added Cu intake (mg) from Cu proteinate

*ALP* alkaline phosphatase, *CER* ceruloplasmin, *Cu/Zn SOD* Cu, Zn-superoxide dismutase, *GSH-Px* glutathione peroxidase, *MDA* malondialdehyde

**Table 11:** Relative bioavailability values (RBV) of copper proteinate (CuPro) based on slope ratios from multiple linear regressions of ADG, FCR, plasma enzyme activities, and jejunal Cu concentrations on daily analyzed dietary Cu intake^1^

Dependent variables	Copper source^2^	Regression coefficients		RBV		*P*-value^3^
		Slope	SE	%	SE	
ADG	TBCC	0.8916	0.18485	100		
	CuPro	1.63437	0.18347	183	34.9	0.0006
FCR	TBCC	−0.003535	0.00024	100		
	CuPro	−0.006068	0.00024	172	17.0	< 0.0001
ALP	TBCC	0.018343	0.00195	100		
	CuPro	0.028563	0.00194	156	12.7	< 0.0001
CER	TBCC	0.11645	0.06564	100		
	CuPro	0.306	0.06538	263	120.1	0.0013
Cu/Zn SOD	TBCC	0.2185	0.17132	100		
	CuPro	0.4489	0.17063	205	126.4	0.1383
GSH-Px	TBCC	0.6748	0.05870	100		
	CuPro	0.7661	0.05846	114	8.0	0.0678
MDA	TBCC	−0.011156	0.00396	100		
	CuPro	−0.014865	0.00394	133	36.9	0.279
Jejunal Cu	TBCC	0.00168	0.00035	100		
	CuPro	0.00349	0.00035	208	38.3	< 0.0001

^a^ Daily dietary analyzed Cu intake = ADFI times analyzed supplemental dietary Cu content (analyzed total Cu content – background Cu content) for each respective Cu source

^b^*TBCC*  tribasic copper chloride, *CuPro* copper proteinate

^c^*P*-value for the difference in slopes among Cu sources

*ALP* alkaline phosphatase, *CER* ceruloplasmin, *Cu/Zn SOD* Cu, Zn-superoxide dismutase, *GSH-Px* glutathione peroxidase, *MDA* malondialdehyde

## Discussion

It is well documented that dietary supplementation with pharmacological amounts of Cu (125–250 mg/kg) increase feed consumption, weight gain, and improve feed efficiency in weanling piglets [3, 24].

In contrast to recent evidence of elevated feed intake [25], which may be attributed to regulation of appetite regulating genes by 175–250 mg/kg Cu as CuSO_4_, our result indicated that either TBCC or CuPro did not affect ADFI. In the present study, greater ADG resulted in an improvement of FCR for pigs during the whole nursery period. The observation that ADG, and final BW were greater for pigs fed diets containing higher levels of TBCC and CuPro than those fed lower Cu corroborates results indicating that supplementation with 50 to 250 mg/kg Cu improved weight gain and feed efficiency in several nursery studies [4, 13, 15, 26]. In a meta-analysis by Braude [27], 250 mg/kg Cu as CuSO_4_ improved ADG on average 8.1% and FCR on average 5.4% compared with no-Cu supplemented pigs. Subsequent analysis also revealed that 250 mg/kg Cu as CuSO_4_ was more effective than 125 mg/kg Cu for performance stimulation in terms of ADG and FCR in growing pigs. Cromwell et al. [3] found 125 mg/kg Cu as CuSO_4_ was approximately 75% as effective as 250 mg/kg Cu as a growth promoter. Due to limited Cu supplementation allowance in nursery diets in China, the maximum Cu concentration of this study was 200 mg/kg Cu as TBCC. Different from the previous findings [3, 25, 28, 29], the addition of 160 mg/kg and 200 mg/kg Cu as TBCC showed no differences in weight gain and feed efficiency, but were higher than pigs fed with less than 80 mg/kg Cu.

Furthermore, CuPro is more effective compared with TBCC as a growth promoter, in stimulating weight gain and feed efficiency for weanling pigs in this experiment, that 80 mg/kg Cu as CuPro was enough to maintain and even promote pig growth compared to pharmaceutical levels of inorganic Cu (160–200 mg/kg). These findings agree with a previous study [15] where CuPro was more effective than CuSO_4_ and even 25 or 50 mg/kg Cu as CuPro had a higher ADG compared with 250 mg/kg Cu as CuSO_4_. This is extremely important when new regulations were applied in different countries and regions, for example, China reduced maximum allowed total Cu in nursery feed from 200 to 125 mg/kg in 2018. Under the current circumstances, regardless of whether TBCC is more effective than CuSO_4_ or not, it is unlikely to provide maximal Cu for optimal pig growth as either TBCC or CuSO_4_. Lower amounts of organic Cu with greater bioavailability such as CuPro could be an alternative to pharmaceutical concentrations of inorganic Cu for growth promotion under the new regulations as well as reducing Cu excretion at the same time. Another advantage of organic Cu could be less negative antagonisms and less interactions with other feed ingredients such as phytase [6, 30].

The mechanisms for growth promoting effects observed from higher than routine levels of Cu are largely unknown. A recent study [31] suggested that the growth-stimulating action of high TBCC was unlikely due to increased digestibility of gross energy or fat/ether extract, which was reported by Luo and Dove [32]. Instead, the growth-promoting effects could be attributed to a positive effect of Cu on the intestinal health of pigs, or an altered microbiota profile [33, 34]. Similar evidence also revealed that the antibacterial function of high Cu in the lumen have a positive effect on growth promotion possibly through alteration in the community structure of microorganisms in the cecum and colon [35, 36]. In the present study, a slightly reduced frequency of diarrhea was also observed in pigs fed diets containing higher levels of Cu, regardless of the Cu sources, which might contribute to a better performance.

There are contradictory studies regarding the efficacy and bioavailability of organic and inorganic sources of Cu in swine diets. In previous studies, when feeding at the same concentrations of Cu, Cu lysine complex has shown to be either as effective [37], or more effective [38] than CuSO_4_, while Cu Polysaccharide [39] was less effective than CuSO_4_, in terms of improving nursery pig growth performance. However, others have reported that feeding a lower concentration of Cu Proteinate in nursery diets resulted in similar or increased growth response of pigs [15]. These variable observations could be partially attributed to inherent chemical variations among different mineral sources resulting in differences in efficacy, bioavailability and possibly different routes of absorption and utilization [40]. The lack of significant growth response to Cu sources at higher concentration (160 mg/kg) in the present study could also be explained for the above variability. Differences were not always observed between CuPro and TBCC at the same supplementation levels, but this did not mean similar efficacy between these two sources. Within the range of 0 to 80 mg/kg Cu, a clear significant improvement in efficacy and bioavailability was found in CuPro compared with TBCC. Therefore, it’s better to determine the right range or concentrations to compare efficacy and bioavailability for different mineral sources.

Plasma and serum concentrations, and tissue retentions of minerals are usually selected to measure mineral utilization in animals [20, 41]. The Cu concentrations in pig plasma in our experiment are consistent with Roof and Mahan [10], in which study plasma Cu was not affected by adding dietary Cu at 250 mg/kg or less compared with control pigs. However, a linear response in plasma Cu concentration with increasing dietary Cu lysine [38] or CuPro [15] in a balance study was observed. The lack of differences among treatments or sources indicates that plasma Cu was not suitable for estimating the Cu requirements of piglets and relative bioavailability of different Cu sources. Also, no alteration of dietary copper levels and sources on plasma Zn and Fe concentrations in the present experiment is in accordance with previous reports for nursery pig studies [10, 15].

Interestingly, a reduction in liver Zn concentration appeared in pigs fed high levels of TBCC rather than pigs fed with CuPro. Interactions and antagonisms between Zn and Cu are well documented. The reason for the reduction in liver Zn for TBCC pigs may be due to antagonism and competition for absorption and transport [29], while there is less antagonism for liver Zn in CuPro pigs since CuPro may be absorbed and utilized through the PepT1 transporter [40]. No similar responses were found in Fe concentrations in all tissues examined, such as liver and small intestinal mucosa. The reduced liver Zn concentrations and unaltered Fe concentrations in tissues of TBCC pigs are in contrast with the results reported by Huang et al. [29] and Fry et al. [28], where liver Zn concentration was greater, but liver Fe concentration was lower in TBCC pigs compared with the control. However, in an organic Cu study, Zhao et al. [4] observed no interaction among minerals, and that dietary Cu had no effect on liver Fe and Zn concentrations. These discrepancies between different studies may be attributed to differences in the ages of pigs, Cu supplementation forms and levels, trial durations, as well as ingredients used in feed formula, among others.

Bile Cu, liver Cu, and intestinal Cu concentrations were all elevated significantly with increasing levels of dietary Cu regardless of their sources. Cu concentrations in these tissues are frequently measured because Cu was metabolized and rich in these tissues. Our findings are in agreement with several previous studies that Cu concentrations in bile, liver, duodenum and jejunum [28, 42] were increased by high Cu supplementation. Agreed with our present study, investigators also found that mucosal Cu concentrations in the duodenum and proximal jejunum were affected by Cu source [29]. Increased Cu retention in liver, duodenum and jejunum mucosa, as well as decreased Cu concentrations in feces for pigs fed with more than 80 mg/kg CuPro compared with corresponding concentrations of TBCC suggested better absorption and utilization of Cu proteinates at higher inclusion rates. Interestingly, bile Cu was more sensitive to dietary Cu levels in the form of CuPro compared with TBCC in our experiment. A previous study suggested a likely hypothesis that intravenous injected Cu histidinate secreted to the bile and bypass the lumen, might have a similar impact on bacteria populations in the gastrointestinal tract [43]. Together with our results, this data may explain that CuPro is more effective than TBCC as a growth promoter and anti-diarrhea agent at lower supplementation levels.

Copper is essential to several key enzymes for normal metabolism, but Cu ions can also act as a prooxidant in diets when supplemented in high levels at 150–250 mg/kg, resulting in oxidation and catalyzing the formation of hydroxyl radicals. Elevated MDA concentrations, a widely used indicator of lipid peroxidation, were reported in liver [44] and duodenal mucosa [29] when pigs were fed higher Cu diets. In our study, MDA concentrations in pig plasma decreased linearly as dietary Cu increased. This is partially supported by the enhanced concentrations of antioxidant enzymes, such as Cu/Zn SOD and GSH-Px in the plasma for pigs fed with higher TBCC or CuPro diets, which consists with our previous study which suggested that enzyme activities in the liver were higher in pigs fed with commercial levels of trace minerals compared with those fed no trace minerals [19]. In a poultry study, greater prooxidant activity was observed in the CuSO_4_ group than those fed with TBCC [45]. Another study also confirmed that TBCC caused less oxidative stress in the small intestine when fed at 225 mg/kg Cu diet using a pig model [28]. In our study, lower MDA concentrations in CuPro compared with TBCC in a dose-dependent manner suggested that strongly bonded Cu to small peptides in CuPro allowed for less liberation of Cu ions resulting in far less potential for reactivity and less reactive oxygen species generation which lead to less oxidation in plasma and thus more stable under the changing pH conditions in the gastrointestinal tract than TBCC.

Copper recommendation for neonatal pigs was based on outdated research done with inorganic forms of Cu using purified diets [46], in which deficiency responses were well defined [47], however, optimal levels for performance were largely unknown. What’s more, commercial production brings more stress than experimental conditions, and enzyme introduction [30, 48], feed processing, and even Cu sources can all alter mineral utilization and thus lead to different Cu requirements for optimal growth, retention or immunity. The efficacy of organic Cu in animals, compared with inorganic Cu, has not been consistent. In some previous studies, Cu in organic proteinate or sulfate was equally bioavailable for broilers fed with either 125 or 250 mg/kg dietary Cu [49], while Cu proteinate was more effective to increase growth performance of 6 kg pigs housed in pens, but not in 11 kg pigs housed individually in metabolism pens [15]. Therefore, it is better to determine some sensitive criteria to evaluate the optimal Cu requirements of different sources for different purpose, such as growth enhancement or immunity.

The choice of criteria used to define requirements is of critical importance, since nutrient recommendation will clearly vary on the criterion used to define nutrient adequacy. Growth performance and tissue retention are frequently used indices for the evaluation of Cu requirements for pigs. Indeed, growth parameters such as ADG and FCR, and bile Cu were very sensitive criteria to assess dietary Cu supplementation when broken-line analysis was used in the present study. An additional 141 or 120 mg/kg Cu as TBCC would suffice for optimal ADG or FCR of piglets fed with a corn-soybean meal-based diet for 38 days after weaning, whereas 80 or 74 mg/kg Cu as CuPro would be enough for the same indices, respectively. To maintain maximum bile Cu concentration, 93 mg/kg Cu as TBCC and 69 mg/kg Cu as CuPro are needed to add to the basal corn-soybean diet. Besides growth parameters and bile Cu retention, the present study also demonstrates some blood biomarkers such as Cu/Zn SOD, CER, GSH-Px, MDA and ALP, as new and sensitive criteria for estimating the Cu optimization status of pigs from 7 kg to 20 kg of weight. Similarly, SOD activity and its mRNA levels in the heart were reported to be a quicker and more consistent index for Mn requirement estimation in broilers from 1 to 21 days of age [50]. Serum ALP activity is suitable for estimating the Zn requirements of brown-egg laying hens from 20 to 40 weeks of age [51]. To achieve the optimal performance of these biomarkers, far less volume was required for CuPro at the same and even higher concentrations when compared with TBCC supplementation, suggesting higher bioavailability for CuPro when using the criteria mentioned above to evaluate Cu efficacy in pigs.

We analyzed the natural Cu content of the basal diet from feed ingredients and found around 9 mg/kg of Cu was provided from diets with no-Cu supplement. Although the bioavailability and digestibility of this Cu from feedstuffs is not known, its contribution should not be ignored in diet formulation. Investigators reported that the natural Cu had an average of 44% digestibility when that basal diet did not contain a trace mineral supplement [52]. However, it was not taken into consideration neither for estimation of TBCC or CuPro requirements nor for later relative bioavailability analysis, due to a lack information on natural forms of Cu. A recent study revealed that phytase could release a large amount of Cu in conventional feedstuffs such as corn and soybean meal based on *in vitro* digestion model [48], thus increasing Cu bioaccessibility of these ingredients. Moreover, super-dosing phytase (20,000 FTU/kg) improved mineral availability and growth performance of weaned pigs [53]. In contrast, feeding high level (up to 250 mg/kg) of Cu from CuSO_4_ to broilers seemed to have a negative effect on phytase, reduced apparent phosphors retention and performance [5, 6]. Another study [54] showed that dietary Zn requirement from Zn proteinate for optimal growth decreased when phytase was supplemented in the maize-soybean meal diet for broiler. These above findings suggested Cu requirement and bioavailability for pigs might vary with and without phytase supplementation in the diet.

The bioavailability of trace minerals is considered as absorbed proportion of ingested element, and conversion of a specific physiological function. The estimation of bioavailability for different Cu sources is highly dependent on the diet type, incorporation of enzymes such as phytase, and sensitive indices selected. Several previous studies reported that organic Cu sources seemed to have equal bioavailability to Cu sulfate [39, 49]. The lack of significant relative bioavailability was unsurprising given the limited grades of Cu supplementation levels, coupled with the small scale of animal numbers. In the current study, we chose ADG, FCR, and several plasma markers as the evaluating criteria because these have been proven to have strong correlation with bioavailability, and these indices increased as added dietary mineral levels increased within a certain range, in accordance with several earlier studies [13, 50, 51] in both swine and poultry. Plasma CER, ALP and jejunal Cu concentrations were significantly higher in the pigs fed diets supplemented with CuPro than in those fed with TBCC within a certain range of levels. Based on these parameters, the relative bioavailability values of CuPro compared to TBCC were 156–263%, suggesting that these indices were sensitive for estimating the bioavailability of Cu sources. In general, these findings are in accordance with previous observations in pigs [4], that organic Cu was more effective than CuSO_4_ and less was required to achieve similar performance or some certain biochemical parameters, while they conflict with a poultry study, which demonstrated no difference in bioavailability between CuPro and CuSO_4_ when supplemented with equivalent inclusion levels of 125 and 250 mg/kg [49].

Most of the bioavailability studies for Cu presented, however, were evaluated from slope ratio analysis of different indicators by supplementation with Cu levels well beyond the requirement. The true Cu bioavailability is influenced by many factors, such as metal-ion interactions, chelating agents, and other dietary components [55]. Very few studies have solely researched copper bioavailability in pigs. Previous bioavailability estimation using purified diets were unsatisfactory as they were too different from practical diets [56], one of the reasons was that most practical diets include phytase or even super-dosing phytase. Several other studies have attempted to use plasma Cu concentrations to determine Cu bioavailability for pigs [15, 18, 38], and while it has been suggested that rather than plasma Cu, liver Cu concentrations are a better indicator for bioavailability between sources [57]. The higher relative bioavailability of CuPro might be partially attributed to the strength of its bonds. Weakly bonded inorganic and organic Cu was reported to result in phytase inhibition [30], vitamin destruction and increased oxidation in both the feed and absorption process, which may lead indirectly to relatively lower bioavailability, Cu retention, and even growth performance in animals. An experiment working with the jejunal tissue of weanling pigs indicated that copper absorption and uptake occurred through different routes between CuPro and CuSO_4_ [40]. Rather than stemming from a large proportion of passive ion flux with copper sulfate, copper proteinate can be absorbed by passive diffusion, active transport, or through absorption while still attached to a short peptide through the PepT1 pathway. Together, these might also help to explain differences in bioavailability between these two types of copper sources. CuPro would be more stable and better able to resist interferences from phytochemicals, pH or other active ligands in the digestive tract, and could deliver to the absorptive site in the small intestine in a better-protected form, thus contributing to more Cu absorption and higher bioavailability.

## Conclusions and implications

In summary, copper proteinate is more effective compared with tribasic copper chloride, and a lesser volume is required to maintain optimal performance, tissue copper status and enzyme activity due to less potential for antagonism and higher bioavailability. With the current limited copper allowance in pig diets, reduced levels of copper proteinate could be an alternative to pharmaceutical inorganic copper to achieve growth promoting effects while reducing environmental copper excretion at the same time. These results suggest that regulations of recommendation, maximum allowance and excretion should take mineral forms into consideration.

It is worth mentioning that the pigs were fed the same diets throughout the nursery phase in the present study. These results should be carefully used when compared with other trials that two or three phases feeding is used. Another limitation is that plasma and tissues samples were only collected at the end of this study, and no measurements was collected at the beginning and in between, which means individual variation at the beginning of this trial was not accounted for. These results from plasma and tissue parameters could only represent the results of pigs at 20 kg BW instead of during the whole experimental period. Further research should focus on systematic investigations of a wide range of copper-containing compounds, enzymes and copper-related transporters at both protein and mRNA levels. Interactions with dietary components such as feed enzymes and vitamins as well as intestinal microbiomes are also required to elucidate the underlying mechanism between copper sources.

## Supplementary information

**Additional file 1: Table S1.** Effect of amount and source of dietary copper on growth performance and diarrhea frequency of weanling pigs. **Table S2.** Effect of amount and source of dietary copper on plasma, bile, intestinal, and liver copper concentrations of weanling pigs. **Table S3.** Effect of amount and source of dietary copper on plasma, intestinal, and liver Zn and Fe concentrations of weanling pigs. **Table S4.** Effect of amount and source of dietary copper on fecal micromineral excretion of weanling pig (mg/kg, as air-dry basis). **Table S5.** Effect of amount and source of dietary copper on plasma enzyme activities and malondialdehyde (MDA) concentrations of weanling pigs.

## Data Availability

All data generated or analyzed in this study is available from the corresponding authors upon reasonable request.

## Abbreviations

ADFI: Average daily feed intake
ADG: Average daily gain
ALP: Alkaline phosphatase
CER: Ceruloplasmin
Cu-Pro: Copper proteinate
Cu/Zn SOD: Cu, Zn-superoxide dismutase
FCR: Feed conversion ratio
GSH-Px: Glutathione peroxidase
MDA: Malondialdehyde
T-AOC: Total antioxidant capability
TBCC: Tribasic copper chloride
