# Supplementary material for: Optimal dietary copper requirements and relative bioavailability for weanling pigs fed either copper proteinate or tribasic copper chloride
Source: J Anim Sci Biotechnol. 2020 May 22;11:54. doi: 10.1186/s40104-020-00457-y (PMC7243316; doi:10.1186/s40104-020-00457-y)
Supplement: Supplementary file 1 — Additional file 1: Table S1. Effect of amount and source of dietary copper on growth performance and diarrhea frequency of weanling pigs. Table S2. Effect of amount and source of dietary copper on plasma, bile, intestinal, and liver copper concentrations of weanling pigs. Table S3. Effect of amount and source of dietary copper on plasma, intestinal, and liver Zn and Fe concentrations of weanling pigs. Table S4. Effect of amount and source of dietary copper on fecal micromineral excretion of weanling pig (mg/kg, as air-dry basis). Table S5. Effect of amount and source of dietary copper on plasma enzyme activities and malondialdehyde (MDA) concentrations of weanling pigs. [file 40104_2020_457_MOESM1_ESM.docx]

Table S1 Effect of amount and source of dietary copper on growth performance and diarrhea frequency of weanling pigs

|  | Cu level, mg/kg | BW d 0, kg | BW d 38, kg | ADG, g/d | ADFI, g/d | FCR, g/g | Diarrhea frequency^1^, % |
| --- | --- | --- | --- | --- | --- | --- | --- |
| NC | 0 | 7.30 | 18.66 | 298.9^b^ | 492.6 | 1.65^a^ | 43.6^a^ |
| TBCC | 5 | 7.43 | 18.83 | 300.1^b^ | 497.4 | 1.66^a^ | 44.0^a^ |
|  | 10 | 7.40 | 18.91 | 302.8^b^ | 501.5 | 1.66^a^ | 43.4^a^ |
|  | 20 | 7.43 | 18.95 | 303.2^b^ | 494.8 | 1.63^a^ | 41.6^a^ |
|  | 40 | 7.43 | 19.48 | 317.2^b^ | 500.0 | 1.58^c^ | 38.4^ab^ |
|  | 80 | 7.23 | 19.82 | 331.2^ab^ | 504.5 | 1.52^d^ | 35.8^b^ |
|  | 160 | 7.27 | 20.88 | 358.1^a^ | 513.0 | 1.43^e^ | 36.0^b^ |
|  | 200 | 7.33 | 20.87 | 356.2^a^ | 519.9 | 1.46^e^ | 35.2^b^ |
| CuPro | 5 | 7.41 | 18.87 | 301.5^b^ | 494.4 | 1.64^a^ | 43.0^a^ |
|  | 10 | 7.44 | 19.01 | 304.4^b^ | 492.9 | 1.62^ab^ | 44.0^a^ |
|  | 20 | 7.37 | 19.24 | 312.4^b^ | 497.6 | 1.59^bc^ | 40.0^ab^ |
|  | 40 | 7.47 | 19.97 | 328.9^ab^ | 502.3 | 1.53^d^ | 39.8^ab^ |
|  | 80 | 7.33 | 21.18 | 364.6^a^ | 518.1 | 1.42^e^ | 32.8^c^ |
|  | 160 | 7.45 | 21.13 | 359.9^a^ | 513.1 | 1.43^e^ | 35.2^b^ |
| Pooled SE |  | 0.34 | 0.83 | 7.56 | 11.87 | 0.009 | 1.86 |
| *P* value^2^ |  | 0.999 | 0.275 | <0.001 | 0.832 | <0.001 | <0.001 |
| Cu source | TBCC | 7.37 | 19.48 | 318.8^b^ | 501.9 | 1.58^a^ | 40.0 |
|  | CuPro | 7.42 | 19.89 | 328.6^a^ | 503.0 | 1.54^b^ | 39.1 |
| Pooled SE |  | 0.14 | 0.35 | 3.24 | 5.07 | 0.003 | 0.96 |
| Cu level, mg/kg | 5 | 7.43 | 18.85 | 300.8^b^ | 495.9 | 1.65^a^ | 43.5^a^ |
|  | 10 | 7.42 | 18.95 | 303.6^b^ | 497.5 | 1.64^a^ | 43.7^a^ |
|  | 20 | 7.40 | 19.10 | 307.8^b^ | 496.3 | 1.61^b^ | 40.8^sb^ |
|  | 40 | 7.45 | 19.70 | 323.1^b^ | 501.9 | 1.55^c^ | 39.1^b^ |
|  | 80 | 7.28 | 20.48 | 347.9^a^ | 510.9 | 1.47^d^ | 34.2^c^ |
|  | 160 | 7.37 | 21.02 | 359.0^a^ | 512.8 | 1.43^e^ | 35.6^c^ |
| Pooled SE |  | 0.25 | 0.61 | 5.62 | 8.78 | 0.006 | 1.46 |
| *P* value^3^ |  |  |  |  |  |  |  |
| Cu source |  | 0.808 | 0.418 | 0.035 | 0.869 | <0.001 | 0.114 |
| Cu level |  | 0.998 | 0.096 | <0.001 | 0.595 | <0.001 | 0.032 |
| Source × Level |  | 0.999 | 0.972 | 0.317 | 0.963 | <0.001 | 0.675 |

NC = negative control, TBCC = tribasic copper chloride, CuPro = copper proteinate.

^1^ Diarrhea score = 5, watery diarrhea, 4, severe diarrhea, 3, mild diarrhea, 2, moist feces, and 1, normal feces. Frequency of diarrhea was calculated by adding all days for a pig with a score of 3 or greater. Frequency = numbers of pigs with diarrhea / (numbers of pigs × the number of days assessing diarrhea scores) × 100%, the number of days assessing diarrhea scores = 9.

^2^ *P* value from ANOVA to compare all 14 treatments.

^3^ *P* value from two-way ANOVA to compare source or level effect, and their interaction.

^a-e^ Means with different superscripts within a column differ (*P <* 0.05).

Table S2 Effect of amount and source of dietary copper on plasma, bile, intestinal, and liver copper concentrations of weanling pigs

|  | Cu level, mg/kg | Plasma, μg/mL | BW Bile, μg/mL | Liver, mg/kg | Duodenum, mg/kg | Jejunum, mg/kg |
| --- | --- | --- | --- | --- | --- | --- |
| NC | 0 | 1.4 | 0.92^fg^ | 23.43^c^ | 2.80^de^ | 5.01^cd^ |
| TBCC | 20 | 1.41 | 0.89^g^ | 23.58^c^ | 2.77^e^ | 4.97^d^ |
|  | 40 | 1.41 | 1.95^d^ | 24.12^c^ | 2.80^de^ | 5.05^bcd^ |
|  | 80 | 1.39 | 2.87^c^ | 24.00^c^ | 2.89^d^ | 5.07^b^ |
|  | 160 | 1.38 | 3.23^b^ | 32.96^b^ | 3.41^b^ | 5.15^b^ |
|  | 200 | 1.36 | 3.2^b^ | 33.92^b^ | 3.38^b^ | 5.28^a^ |
|  | 20 | 1.42 | 1.10^f^ | 23.58^c^ | 2.80^de^ | 5.09^bc^ |
|  | 40 | 1.40 | 2.36^d^ | 23.77^c^ | 2.83^de^ | 5.10^bc^ |
| CuPro | 80 | 1.39 | 3.21^c^ | 31.91^b^ | 3.06^c^ | 5.12^b^ |
|  | 160 | 1.38 | 3.44^a^ | 37.01^a^ | 3.58^a^ | 5.30^a^ |
| Pooled SE |  | 0.02 | 0.03 | 0.54 | 0.02 | 0.02 |
| *P* value^1^ |  | 0.333 | <0.001 | <0.001 | <0.001 | <0.001 |
| Cu source | TBCC | 1.40 | 2.23^b^ | 26.16^b^ | 2.97^b^ | 5.06^b^ |
|  | CuPro | 1.40 | 2.48^a^ | 29.07^a^ | 3.07^a^ | 5.15^a^ |
| Pooled SE |  | 0.01 | 0.02 | 0.25 | 0.01 | 0.01 |
| Cu level, mg/kg | 20 | 1.42 | 0.99^d^ | 23.58^c^ | 2.78^c^ | 5.03^c^ |
|  | 40 | 1.41 | 2.15^c^ | 23.95^c^ | 2.82^c^ | 5.08^bc^ |
|  | 80 | 1.39 | 2.94^b^ | 27.95^b^ | 2.97^b^ | 5.09^b^ |
|  | 160 | 1.38 | 3.34^a^ | 34.99^a^ | 3.49^a^ | 5.22^a^ |
| Pooled SE |  | 0.01 | 0.03 | 0.35 | 0.02 | 0.01 |
| *P* value^2^ |  |  |  |  |  |  |
| Cu source |  | 0.943 | <0.001 | <0.001 | <0.001 | <0.001 |
| Cu level |  | 0.146 | <0.001 | <0.001 | <0.001 | <0.001 |
| Source × Level | | 0.923 | 0.020 | <0.001 | 0.001 | 0.056 |

NC = negative control, TBCC = tribasic copper chloride, CuPro = copper proteinate.

^1^ *P* value from ANOVA to compare all 10 treatments.

^2^ *P* value from two-way ANOVA to compare effects of source, level and their interaction.

^a-g^ Means with different superscripts within a column differ (*P <* 0.05).

Table S3 Effect of amount and source of dietary copper on plasma, intestinal, and liver Zn and Fe concentrations of weanling pigs

|  | Cu level, mg/kg | Zn | | | |  | Fe | | | |
| --- | --- | --- | --- | --- | --- | --- | --- | --- | --- | --- |
|  |  | Plasma, μg/mL | Liver, mg/kg | Duodenum, mg/kg | Jejunum, mg/kg |  | Plasma, μg/mL | Liver, mg/kg | Duodenum, mg/kg | Jejunum, mg/kg |
| NC | 0 | 1.63 | 315.96^a^ | 109.51 | 113.34 |  | 4.16 | 368.19 | 249.15 | 140.26 |
| TBCC | 20 | 1.63 | 318.83^a^ | 108.66 | 114.16 |  | 4.12 | 365.71 | 248.53 | 140.53 |
|  | 40 | 1.65 | 310.89^ab^ | 110.67 | 114.79 |  | 4.17 | 362.06 | 248.02 | 139.83 |
|  | 80 | 1.64 | 309.09^abc^ | 110.24 | 114.25 |  | 4.27 | 361.25 | 246.48 | 140.47 |
|  | 160 | 1.67 | 287.04^c^ | 110.56 | 114.73 |  | 4.25 | 375.93 | 245.72 | 140.65 |
|  | 200 | 1.66 | 288.11^bc^ | 111.71 | 115.73 |  | 4.34 | 374.49 | 246.92 | 140.18 |
|  | 20 | 1.66 | 317.92^a^ | 111.09 | 116.69 |  | 4.24 | 360.99 | 245.83 | 139.29 |
|  | 40 | 1.67 | 316.48^a^ | 108.41 | 115.64 |  | 4.21 | 366.26 | 246.59 | 140.65 |
| CuPro | 80 | 1.70 | 313.83^a^ | 110.08 | 115.98 |  | 4.27 | 372.85 | 245.59 | 140.37 |
|  | 160 | 1.67 | 305.50^abc^ | 111.12 | 116.22 |  | 4.23 | 372.71 | 246.44 | 140.48 |
| Pooled SE |  | 0.03 | 4.81 | 0.96 | 0.80 |  | 0.15 | 10.32 | 0.94 | 0.47 |
| *P* value^1^ |  | 0.559 | <0.001 | 0.098 | 0.119 |  | 0.996 | 0.968 | 0.124 | 0.596 |
| Cu source | TBCC | 1.65 | 306.46 | 110.27 | 114.48 |  | 4.20 | 366.24 | 246.11 | 140.37 |
|  | CuPro | 1.67 | 312.73 | 110.18 | 116.13 |  | 4.24 | 368.20 | 247.19 | 140.20 |
| Pooled SE |  | 0.01 | 2.43 | 0.50 | 0.24 |  | 0.08 | 5.60 | 0.24 | 0.25 |
| Cu level, mg/kg | 20 | 1.64 | 318.37^a^ | 109.60 | 115.43 |  | 4.18 | 363.35 | 247.18 | 139.91 |
|  | 40 | 1.66 | 313.69^a^ | 109.54 | 115.22 |  | 4.19 | 364.16 | 247.30 | 140.24 |
|  | 80 | 1.67 | 311.46^a^ | 110.16 | 115.11 |  | 4.27 | 367.05 | 246.03 | 140.42 |
|  | 160 | 1.67 | 294.86^b^ | 111.59 | 115.48 |  | 4.24 | 374.32 | 246.30 | 140.56 |
| Pooled SE |  | 0.02 | 3.44 | 0.71 | 0.35 |  | 0.11 |  | 0.34 | 0.35 |
| *P* value^2^ |  |  |  |  |  |  |  |  |  |  |
| Cu source |  | 0.134 | 0.081 | 0.897 | 0.071 |  | 0.758 | 0.806 | 0.059 | 0.627 |
| Cu level |  | 0.645 | <0.001 | 0.175 | 0.865 |  | 0.922 | 0.756 | 0.054 | 0.590 |
| Source × Level | | 0.734 | 0.409 | 0.088 | 0.414 |  | 0.967 | 0.878 | 0.014 | 0.249 |

NC = negative control, TBCC = tribasic copper chloride, CuPro = copper proteinate.

^1^ *P* value from ANOVA to compare all 10 treatments.

^2^ *P* value from two-way ANOVA to compare effects of source, level and their interaction.

^a-c^ Means with different superscripts within a column differ (*P <* 0.05).

Table S4 Effect of amount and source of dietary copper on fecal micromineral excretion of weanling pig (mg/kg, as air-dry basis)

|  | Cu level, mg/kg | Fecal Cu | Fecal Zn | Fecal Fe |
| --- | --- | --- | --- | --- |
| NC | 0 | 121.30^i^ | 5720.19 | 3058.96 |
| TBCC | 20 | 280.10^j^ | 5865.70 | 3051.32 |
|  | 40 | 415.37^f^ | 5729.98 | 2998.86 |
|  | 80 | 656.00^d^ | 5885.98 | 2989.07 |
|  | 160 | 917.14^b^ | 5786.74 | 2953.81 |
|  | 200 | 1353.44^a^ | 5899.92 | 3042.00 |
|  | 20 | 255.82^i^ | 5928.12 | 2963.80 |
|  | 40 | 362.15^g^ | 5760.37 | 2964.34 |
| CuPro | 80 | 600.67^e^ | 5883.88 | 3000.19 |
|  | 160 | 873.10^c^ | 5793.44 | 2976.71 |
| Pooled SE |  | 8.27 | 89.61 | 81.32 |
| *P* value^1^ |  | <0.001 | 0.690 | 0.989 |
| Cu source | TBCC | 567.15^a^ | 5817.10 | 2998.27 |
|  | CuPro | 522.93^b^ | 5841.45 | 2976.26 |
| Pooled SE |  | 4.42 | 45.83 | 42.04 |
| Cu level, mg/kg | 20 | 267.96^d^ | 5896.91 | 3007.56 |
|  | 40 | 388.76^c^ | 5745.17 | 2981.60 |
|  | 80 | 628.33^b^ | 5884.93 | 2994.63 |
|  | 160 | 895.12^a^ | 5790.09 | 2965.26 |
| Pooled SE |  | 6.25 | 64.82 | 59.45 |
| *P* value^2^ |  |  |  |  |
| Cu source |  | <0.001 | 0.710 | 0.715 |
| Cu level |  | <0.001 | 0.300 | 0.963 |
| Source × Level | | 0.304 | 0.985 | 0.910 |

NC = negative control, TBCC = tribasic copper chloride, CuPro = copper proteinate.

^1^ *P* value from ANOVA to compare all 10 treatments.

^2^ *P* value from two-way ANOVA to compare effects of source, level and their interaction.

^a-j^ Means with different superscripts within a column differ (*P <* 0.05).

Table S5 Effect of amount and source of dietary copper on plasma enzyme activities and malondialdehyde (MDA) concentrations of weanling pigs

|  | Cu level, mg/kg | ALP, U/100mL | CER,  U/mL | Cu/Zn SOD, U/mL | GSH-Px, U/mL | MDA, nmol/mL | T-AOC, U/mL |
| --- | --- | --- | --- | --- | --- | --- | --- |
| NC | 0 | 1.68^e^ | 57.34^e^ | 121.76^b^ | 730.31^f^ | 2.36^a^ | 0.61 |
| TBCC | 20 | 1.78^e^ | 73.12^d^ | 135.09^ab^ | 729.40^f^ | 2.31^ab^ | 0.62 |
|  | 40 | 2.05^cd^ | 76.26^cd^ | 134.96^ab^ | 741.17^cd^ | 2.17^abc^ | 0.63 |
|  | 80 | 2.36^b^ | 80.12^bcd^ | 139.82^ab^ | 750.61^ab^ | 1.81^c^ | 0.69 |
|  | 160 | 2.94^a^ | 83.27^abc^ | 146.71^a^ | 748.72^b^ | 1.78^c^ | 0.71 |
|  | 200 | 3.00^a^ | 85.90^ab^ | 145.34^ab^ | 746.85^bc^ | 1.84^c^ | 0.71 |
|  | 20 | 1.91^de^ | 78.91^bcd^ | 133.97^ab^ | 732.63^ef^ | 2.11^abc^ | 0.65 |
|  | 40 | 2.25^cd^ | 83.67^abc^ | 140.68^ab^ | 738.90^de^ | 1.89^abc^ | 0.66 |
| CuPro | 80 | 2.80^a^ | 85.35^ab^ | 149.85^a^ | 756.35^a^ | 1.79^c^ | 0.71 |
|  | 160 | 3.05^a^ | 88.74^a^ | 154.82^a^ | 757.54^a^ | 1.75^c^ | 0.72 |
| Pooled SE |  | 0.06 | 1.66 | 5.18 | 1.55 | 0.10 | 0.05 |
| *P* value^1^ |  | <0.001 | <0.001 | 0.003 | <0.001 | <0.001 | 0. 792 |
| Cu source | TBCC | 2.28^b^ | 78.19^b^ | 139.15 | 742.47^b^ | 2.02 | 0.66 |
|  | CuPro | 2.50^a^ | 84.17^a^ | 144.83 | 746.38^a^ | 1.88 | 0.68 |
| Pooled SE |  | 0.03 | 0.88 | 2.67 | 0.80 | 0.05 | 0.03 |
| Cu level, mg/kg | 20 | 1.85^d^ | 76.02^c^ | 134.52^b^ | 730.95^c^ | 2.21^a^ | 0.63 |
|  | 40 | 2.15^c^ | 79.96^bc^ | 137.83^ab^ | 740.00^b^ | 2.03^ab^ | 0.64 |
|  | 80 | 2.58^b^ | 82.74^ab^ | 144.84^ab^ | 753.55^a^ | 1.80^b^ | 0.70 |
|  | 160 | 3.00^a^ | 86.01^a^ | 150.77^a^ | 753.19^a^ | 1.77^b^ | 0.71 |
| Pooled SE |  | 0.04 | 1.24 | 3.78 | 1.13 | 0.07 | 0.04 |
| *P* value^2^ |  |  |  |  |  |  |  |
| Cu source |  | <0.001 | <0.001 | 0.140 | 0.001 | 0.072 | 0.578 |
| Cu level |  | <0.001 | <0.001 | 0.019 | <0.001 | <0.001 | 0.412 |
| Source × Level | | 0.022 | 0.924 | 0.741 | 0.008 | 0.486 | 0.994 |

ALP: Alkaline phosphatase, CER: ceruloplasmin, Cu/Zn SOD: Cu, Zn-Superoxide dismutase, GSH-Px: glutathione peroxidase, MDA: malondialdehyde, T-AOC: total antioxidant capability.

NC = negative control, TBCC = tribasic copper chloride, CuPro = copper proteinate.

^1^ *P* value from ANOVA to compare all 10 treatments.

^2^ *P* value from two-way ANOVA to compare effects of source, level and their interaction.

^a-f^ Means with different superscripts within a column differ (*P <* 0.05).
